# Supragingival *Actinomyces naeslundii* aggravates metabolic dysfunction-associated fatty liver disease via the oral–gut axis

**DOI:** 10.1080/20002297.2026.2639208

**Published:** 2026-03-07

**Authors:** Ye Tu, Zhanyi Chen, Meiling Jing, Wenduo Tan, Danni Huang, Jin Xu, Min Wang, Hao Li, Yueyi Yang, Xiaoyu Liu, Xuchen Hu, Yihuai Pan, Chenguang Niu, Zhengwei Huang

**Affiliations:** a Department of Endodontics, Shanghai Ninth People's Hospital, Shanghai Jiao Tong University School of Medicine, Shanghai, China; b College of Stomatology, Shanghai Jiao Tong University, Shanghai, China; c National Clinical Research Center for Oral Diseases, National Center for Stomatology, Shanghai, China; d Shanghai Key Laboratory of Stomatology, Shanghai, China; e Institute of Stomatology, School and Hospital of Stomatology, Wenzhou Medical University, Wenzhou, China; f Department of Endodontics, School and Hospital of Stomatology, Wenzhou Medical University, Wenzhou, China; g State Key Laboratory of Molecular Engineering of Polymers, Fudan University, Shanghai, China

**Keywords:** Metabolic dysfunction-associated fatty liver disease, oral microbiota, *Actinomyces naeslundii*, oral–gut axis, metagenomics

## Abstract

**Background:**

Metabolic dysfunction-associated fatty liver disease (MAFLD) is the most prevalent chronic liver disease but lacks effective therapies. Oral microbial dysbiosis is closely associated with metabolic dysfunction.

**Objective:**

This study aimed to delineate MAFLD-specific oral microbiota signatures and identify diagnostic biomarkers.

**Design:**

Supragingival plaque samples from 21 patients with MAFLD and 20 healthy individuals were subjected to metagenomic sequencing. Potential oral biomarkers were identified bioinformatically and further validated using a MAFLD mouse model.

**Results:**

Patients with MAFLD exhibited significantly reduced supragingival microbial diversity, altered composition, and enhanced consortial interactions compared to healthy individuals. Seven resident oral species were identified as candidate biomarkers. Among these, *Actinomyces naeslundii* was notably enriched in the oral cavity of patients with MAFLD and strongly correlated with clinical indices. *In vivo* experiments further demonstrated that the oral administration of *A. naeslundii* significantly aggravated MAFLD phenotypes and induced gut dysbiosis in mice fed a high-fat diet.

**Conclusion:**

This study reveals a potential link between the oral microbiota and MAFLD. Specifically, the excessive enrichment of the oral resident bacterium *A. naeslundii* is associated with the MAFLD progression in mice.

## Introduction

The human oral cavity hosts the second most diverse microbial community in the human body. This complex and dynamic ecosystem comprises bacteria, archaea, fungi and viruses existing predominantly in a state of symbiotic homoeostasis with the host [1,2]. However, this delicate balance is susceptible to disruption, which can precipitate local pathologies such as periodontitis and dental caries [3]. Critically, the oral cavity is not an isolated organ but rather a well-documented reservoir of microorganisms and inflammatory mediators that can exert influences far beyond the teeth and gingiva [4]. The loss of integrity of the gingival sulcus, after being compromised by inflammatory periodontitis, creates a pathological portal enabling oral bacteria, their metabolites and pro-inflammatory cytokines to enter the systemic circulation via bacteremia during chewing or swallowing. Our previous study has revealed a potential ‘oral–gut axis’ that links periodontitis with insulin resistance [5]. Given that nearly 1 × 10^12^ oral bacteria are continuously ingested and reach the gut, this continual seeding positions the oral microbiome as a plausible yet underexplored contributor to systemic inflammation and distant organ pathologies [6].

Metabolic dysfunction-associated fatty liver disease (MAFLD) is a globally prevalent chronic liver condition characterised by hepatic steatosis and metabolic dysregulation and has an estimated global prevalence of 25% [7–9]. In MAFLD, metabolic dysfunction has a central role in driving disease progression and promoting systemic extrahepatic complications [10]. Although less than 10% of patients with MAFLD develop severe outcomes, including cirrhotic complications and hepatocellular carcinoma, within 10–20 years after diagnosis, the high prevalence of the disease leads to a substantial absolute number of severe cases. The pathogenesis is multifactorial, driven by a complex interplay among genetic susceptibility, insulin resistance, lipotoxicity, oxidative stress and chronic inflammation [11,12]. Despite extensive research efforts, the detailed mechanisms underlying disease progression remain incompletely understood, leading to a lack of approved pharmacotherapies. This underscores the urgent need to explore novel pathogenic factors and therapeutic strategies that extend beyond conventional metabolic pathways. In recent years, growing studies have increasingly highlighted the comorbidity between oral dysbiosis and MAFLD, indicating that altered oral microbiota may exacerbate systemic inflammation and liver steatosis through the oral–gut–liver axis [13,14].

Proposed mechanisms often mirror those of the gut‒liver axis, thereby implicating a basis in systemic inflammation fuelled by circulating oral-derived pathogens and mediators [15,16]. Specific keystone periodontal pathogens, such as *Porphyromonas gingivalis*, have been experimentally shown to exacerbate hepatic steatosis and inflammation in animal models [17,18]. Research to date has focused predominantly on the effects of periodontitis and its associated pathogens in MAFLD, whereas the functions of the broader community of oral resident flora have been largely unexplored. The lack of a comprehensive, unbiased characterisation of the role of oral resident flora in MAFLD leaves a critical question unanswered: is oral dysbiosis a mere bystander, a modifier, or an active contributor to MAFLD pathogenesis? This issue warrants deeper investigation.

To address this critical gap, the current study aimed to systematically characterise the oral microbiota of patients with MAFLD and to evaluate the potential roles of candidate bacteria in disease pathogenesis. A clinical cohort consisting of 21 patients with MAFLD and 20 healthy controls was enroled, and supragingival plaque samples were collected to investigate alterations of oral microbiota. Metagenomic sequencing combined with bioinformatics analysis revealed distinct oral microbiota profiles in patients with MAFLD and identified seven resident bacteria, including *Actinomyces naeslundii* (*A. naeslundii*), as potential oral biomarkers that effectively distinguished the patients with MAFLD from healthy individuals. Further *in vivo* experiments in mice were conducted to investigate the pathogenic role of key candidate biomarker. Oral overabundance of *A. naeslundii* significantly exacerbated MAFLD in mice fed by a high-fat diet (HFD). Overall, this study elucidated the close association between the oral microbiota and MAFLD and highlighted the functional contribution of specific oral commensal bacteria to disease progression.

## Materials and methods

### Clinical cohort enrolment and sample collection

Informed consent was obtained from all participants prior to their enrolment. A total of 21 patients diagnosed with MAFLD were recruited based on the inclusion and exclusion criteria. Twenty healthy controls were enroled by matching for age. The exclusion criteria included the following: the presence of other liver diseases, including viral hepatitis, autoimmune hepatitis and hepatolenticular degeneration; hepatic steatosis induced by drugs, such as tamoxifen, amiodarone, valproate, methotrexate and glucocorticoids; other factors that may cause hepatic steatosis, including long-term total parenteral nutrition, inflammatory bowel disease, coeliac disease, hypothyroidism, Cushing's syndrome, lipoprotein deficiency, lipid-atrophic diabetes and others; the use of lipid-lowering drugs in the 6 months preceding enrolment; type 1 or type 2 diabetes; current oral diseases, including untreated oral abscess, precancerous lesions, oral cancer, oral fungal infection, missing more than eight teeth, periodontitis and others; the use of probiotics, antifungal drugs; and other conditions, including pregnant or lactating women, long-term heavy smoking, use of antibiotics for more than 5 days within the preceding 6 months, severe acute episode of a systematic disease, abnormal thyroid function, familial hyperlipidemia and others.

Demographic information (age, gender, weight and height) and clinical parameters (total cholesterol (TC), total triglyceride (TG), low-density lipoprotein cholesterol (LDL-C), high-density lipoprotein cholesterol (HDL-C), alanine aminotransferase (ALT), aspartate aminotransferase (AST), gamma glutamyl transpeptidase (GGT), fasting blood glucose (FBG), fasting serum insulin (FSI), glycated haemoglobin A1c (HbA1c), white blood cell (WBC) and plasma high-sensitivity C-reactive protein (CRP)) were collected by trained staff. Supragingival plaque samples were freshly collected from 41 participants as previously described [19]. The participants were instructed to avoid oral hygiene practices the night prior to sample collection. Supragingival plaque samples were collected in the morning before eating by using curettes and preserved in 2-mL sterile tubes (Eppendorf, Hamburg, Germany). All samples were transported to the laboratory on ice packs within 2 h and stored at −80 °C.

### Metagenome sequencing and data processing

Genomic DNA from supragingival plaque was extracted using the QIAamp DNA Mini Kit (Qiagen, Valencia, CA, USA). Metagenomic sequencing was performed on an Illumina NovaSeq 6000 (Illumina Inc., San Diego, CA, USA) at Majorbio Bio-Pharm Technology Co., Ltd. (Shanghai, China). The raw reads were quality-trimmed and filtered using fastp (https://github.com/OpenGene/fastp, version 0.20.0). The quality-filtered reads were assembled into contigs using MEGAHIT (https://github.com/voutcn/megahit, version 1.1.2). Open reading frames (ORFs) from each assembled contig were predicted using Prodigal (https://github.com/hyattpd/Prodigal, version 2.6.3). The predicted ORFs with a length ≥ 100 bp were retrieved and translated into amino acid sequences using the NCBI translation table. A non-redundant gene catalogue was constructed using CD-HIT (http://weizhongli-lab.org/cd-hit/, version 4.7) with 90% sequence identity and 90% coverage. The gene abundance for a certain sample was estimated by SOAPaligner (https://github.com/ShujiaHuang/SOAPaligner, version soap2.21 release) with 95% identity. The best-hit taxonomy of non-redundant genes was determined by aligning them against the NCBI NR database by DIAMOND (http://ab.inf.uni-tuebingen.de/software/diamond/, version 2.0.13). The sequencing data are deposited in the NCBI Short Read Archive database (accession: SRP429021).

### Bacterial strain cultivation

*A. naeslundii* ATCC 19039 was anaerobically cultured in BHI broth (BD Bioscience, San Jose, CA) at 37 °C under a gas mixture of 90% nitrogen, 5% hydrogen and 5% carbon dioxide until it reached a concentration of 1 × 10⁸ CFU/mL. The culture was resuspended to a concentration of 1 × 10^6^ CFU/100 μL in carboxymethyl cellulose (CMC) for oral administration to the mice.

### Animal experiments

Seven-week-old male C57BL/6 J mice (Jiesijie Laboratory Animal Technology Co., Ltd.) were used for the experiments and housed in SPF-level facilities under standard conditions (40–70% air humidity, 22 ± 2 °C ambient temperature and a 12/12 h light/dark cycle). After a 1-week acclimatisation period, the mice were randomly assigned to four groups: chow diet (CD) + vehicle (Veh), high-fat diet (HFD) + Veh, CD + *A. naeslundii* (An) and HFD + An. Mice in the HFD + Veh and HFD + An groups were provided with a HFD (60% fat, 20% protein, 20% carbohydrate; Research Diets, D12492; Jiangsu Xietong Biology Co., Ltd.), while those in the CD + Veh and CD + An groups received a standard chow diet. The mice in the CD + An and HFD + An groups were orally administered 1 × 10^6^ CFU of freshly prepared *A. naeslundii* in 100 μL of CMC solution four times per week. The *A. naeslundii* solution was applied to the oral cavity of the mice by gently swabbing on the teeth, periodontal tissues and mucous membranes. The mice in the CD + Veh and HFD + Veh groups received the 100 μL CMC solution orally, also four times per week.

After eight weeks of experimental treatment, the samples were harvested for analysis. Faecal samples were collected in 1.5-mL sterile Eppendorf tubes and immediately stored at −80 °C. Blood samples were collected from the abdominal aorta under intraperitoneal anaesthesia and centrifuged at 3,000 rpm for 15 min at 4 °C to obtain the serum. Following euthanasia, 2-cm segments of ileum were immediately excised and submerged into 1-mL of TRIzol^®^ reagent (Invitrogen, Carlsbad, CA, USA) for RNA isolation. The liver and mesenteric white adipose tissue (WAT) were dissected, weighed and fixed in 4% paraformaldehyde for 48 h.

### Biochemical analysis and tissue histological analysis

The body weight of the mice was monitored on a weekly basis. Fat gain at week 8 was assessed using the fat index, which was calculated as the ratio of WAT weight to total body weight. Glucose metabolic parameters were evaluated one week prior to euthanasia through FBG and oral glucose tolerance test (OGTT). Following a 6-h fasting period, the mice were given an oral gavage of a glucose load (2 g/kg body weight). Tail vein blood samples were collected before glucose administration (time point 0) and at 15-, 30-, 60- and 120-min post-administration, with blood glucose levels were measured using a glucose metre (Sannuo, China). Serum concentrations of TC, TG, LDL and HDL were measured using assay kits (Jiancheng Bioengineering Institute, China) according to the manufacturer's instructions.

Liver and WAT samples were embedded in paraffin and sliced into 5-μm sections. The tissue sections were processed for hematoxylin and eosin (HE) staining. Liver histopathology was evaluated using the Nonalcoholic Steatohepatitis Clinical Research Network (NASH-CRN) system [20]. The diameter of the adipocyte was measured to assess WAT morphology.

### 16S rRNA gene amplicon sequencing and data processing (mice faecal samples)

Genomic DNA was extracted from mice faecal samples using the E.Z.N.A.® soil DNA Kit (Omega Bio-tek, Norcross, GA, U.S.). The bacterial 16S rRNA genes were amplified using the universal bacterial primers 338F (5′-ACTCCTACGGGAGGCAGCAG-3′) and 806R (5′-GGACTACHVGGGTWTCTAAT-3′). Amplification was performed under the following conditions: initial denaturation at 95 °C for 3 min, followed by 27 cycles of 95 °C for 30 s, annealing at 60 °C for 30 s, extension at 72 °C for 45 s and a single extension at 72 °C for 10 min, and ended at 4 °C (T100 Thermal Cycler PCR thermocycler, BIO-RAD, USA). Purified amplicons were pooled in equimolar, and the DNA library was constructed using the SMRTbell prep kit 3.0 (Pacific Biosciences, CA, USA). Purified SMRTbell libraries were sequenced on the Illumina MiSeq 300PE (Illumina Inc., San Diego, CA, USA) by Majorbio Bio-Pharm Technology Co., Ltd. (Shanghai, China). High-fidelity (HiFi) reads were barcode-identified and length-filtered, and sequences with a length < 1,000 or >1,800 bp were removed. The optimised-HiFi reads were clustered into operational taxonomic units (OTUs) at 97% similarity using UPARSE 7.1. Taxonomic classification was assigned using the RDP Classifier version 2.2 against the 16S rRNA gene database (Silvav138) (confidence threshold 0.7). Sequencing data are deposited in the NCBI Short Read Archive database (accession number: SRP626831).

### Quantitative real-time polymerase chain reaction (qRT-PCR) of gene expression

Total RNA from mice ileum tissue was extracted with TRIzol® reagent (Invitrogen, Carlsbad, CA, USA). Reverse transcription of RNA into cDNA was carried out using the TaKaRa Ex Premier DNA Polymerase (RR370, Takara Bio, Dalian, China). The expression of genes encoding intestinal tight junction proteins, including Occludin, Claudin-1, ZO-1 and MUC-1 was quantified with GAPDH as an internal control. Genomic DNA from mice faecal samples was isolated and purified as described in the section 16S rRNA gene amplicon sequencing and data processing (mice faecal samples). The relative abundance of *A. naeslundii* in faecal samples was measured, with the 16S-univ-1 gene used as an internal control. Relative quantification was performed using the 2^−ΔΔCT^ method. The primer sequences used are provided in Supplemental Table 1.

### Bioinformatics analysis

Microbiome sequencing data (metagenome and 16S rRNA gene sequencing) were processed using the Majorbio Cloud Platform (www.majorbio.com). Alpha diversity was assessed through the Chao, Shannon and Simpson indices. Beta diversity was determined by principal coordinates analysis (PCoA) based on Bray‒Curtis dissimilarity matrices, supplemented by analysis of similarities (ANOSIM) test. Linear discriminant analysis (LDA) effect size (LEfSe) was conducted to identify the differentially abundant taxa that most likely explained the differences across groups, with an LDA score threshold of ≥2. Potential biomarkers were evaluated using a random forest classifier and screened by mean decrease in accuracy. The diagnostic performance of the candidate biomarkers was validated through receiver operating characteristic (ROC) curve analysis, with the area under the curve (AUC) as the evaluation metric. Spearman's correlation analysis was performed to assess the relationship between the oral microbiota and clinical indicators. The co-occurrence network with Spearman's correlation analysis was employed to investigate the community interaction. Statistical comparisons were performed using the Wilcoxon rank-sum test for two groups, while the Kruskal‒Wallis H test with Dunn's post hoc test was employed for multiple group comparisons. All the statistical tests considered *p* < 0.05 as significant, with values reported to at least three decimal places.

### Statistical analysis

All the statistical analyses were performed using GraphPad Prism (GraphPad Software, version 8.0). For comparisons between two groups, either the independent samples *t-*test or Wilcoxon rank-sum test was employed. Multiple group comparisons were analysed using one-way ANOVA followed by Tukey's HSD or Fisher's LSD post hoc test, or the Kruskal‒Wallis H test with Dunn's post hoc test. All the statistical tests considered *p* < 0.05 as significant.

## Results

### Participant characteristics

A total of 41 participants were enroled, comprising 21 patients with MAFLD and 20 healthy individuals. The demographic and clinical characteristics of the cohort are summarised in Table 1 and have previously been reported [19]. No significant differences were observed in sex, age and height between the groups. Among the clinical parameters, higher weight, BMI, serum lipids, blood glucose-associated indicators and inflammatory markers were observed in patients with MAFLD than in healthy individuals.

**Table 1.** Demographic information and clinical parameters of participants.

Characteristics	Healthy control (*n* = 20)	Patients with MAFLD (*n* = 21)	*p-*value
Sex (M/F)	16/4	16/5	1
Age (years)	29.00 ± 8.89	32.43 ± 6.59	0.1778
Height (cm)	172.10 ± 7.59	169.33 ± 6.71	0.2344
Weight (kg)	65.68 ± 8.55	74.81 ± 13.08	0.0142*
BMI (kg/m^2^)	22.09 ± 1.92	25.96 ± 3.27	0.0001***
TC (mmol/L)	4.30 ± 0.64	4.87 ± 1.02	0.0429*
TG (mmol/L)	1.07 ± 0.44	1.82 ± 0.82	0.0010**
LDL-C (mmol/L)	2.59 ± 0.50	3.13 ± 0.91	0.0291*
HDL-C (mmol/L)	1.25 ± 0.24	1.09 ± 0.23	0.0395*
GGT (U/L)	16.40 ± 4.98	35.10 ± 18.93	0.0002***
AST (U/L)	18.10 ± 5.13	28.33 ± 20.88	0.0443*
ALT (U/L)	17.05 ± 9.13	39.33 ± 23.32	0.0004***
FBG (mmol/L)	4.71 ± 0.33	4.85 ± 0.44	0.2652
FSI (mU/L)	5.90 ± 2.18	11.30 ± 5.20	0.0002***
HOMA-IR	1.28 ± 0.48	2.47 ± 1.27	0.0003***
HbA1c	5.18 ± 0.21	5.33 ± 0.28	0.0664
CRP (mg/L)	1.15 ± 1.28	2.11 ± 2.38	0.1296
WBC (×10^9^/L)	5.90 ± 1.12	6.28 ± 1.25	0.3282

Note: Values are presented as mean and standard deviation. Student's *t*-test or the Kruskal–Wallis H test was applied for comparisons of clinical variables between two groups. *p-value < 0.05, **p-value < 0.01 and ***p < 0.001. Abbreviations: BMI, body mass index; TC, total cholesterol; TG, total triglyceride; LDL-C, low-density lipoprotein cholesterol; HDL-C, high-density lipoprotein cholesterol; GGT, gamma glutamyl transpeptidase; AST, aspartate aminotransferase; ALT, alanine aminotransferase; FBG, fasting blood glucose; FSI, fasting serum insulin; HOMA-IR, homoeostatic model assessment for insulin resistance; HbA1c, glycated haemoglobin A1c; CRP, plasma high-sensitivity C-reactive protein and WBC, white blood cell.

### Alterations in the supragingival microbiota in patients with MAFLD

**Figure 1.**
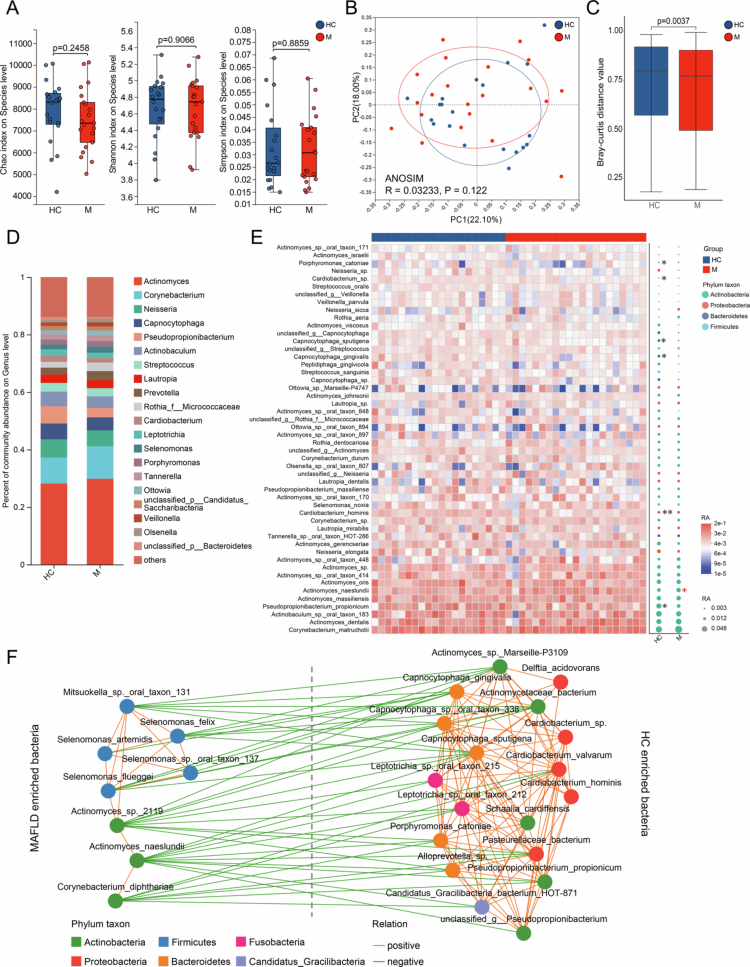
Diversity and composition of supragingival microbiota. (A) Alpha diversity assessed by the Chao, Shannon and Simpson indices at the species level (Wilcoxon rank-sum test). (B) PCoA with ANOSIM test based on Bray‒Curtis distance at the species level. (C) Comparison of beta diversity based on Bray‒Curtis distance at the species level (Wilcoxon rank-sum test). (D) The composition of the supragingival microbiota at the genus level is presented as stacked bar plots. The ‘other’ subcategory included rare species (relative abundance < 0.1%). (E) Clustering heatmaps and bubble diagrams of the relative abundance of the top 50 species. The top row displays colour-coded squares, with blue and red representing samples from the healthy and MAFLD groups, respectively. The colour intensity of heatmap blocks and the size of bubbles reflect the relative abundance of species. The colour of the bubbles denotes the phylum-level taxonomy of the species. Significant differential abundance between groups is indicated by black asterisks (healthy group) and red asterisks (MAFLD group) (Wilcoxon rank-sum test). (F) Co-occurrence network of species with relative abundance >0.1% and differentially enriched in the healthy and MAFLD group. Orange and green edges indicate positive and negative correlations, respectively (Spearman, *p-*value < 0.05, *R* > 0). The colour of the nodes represents the phylum-level taxonomy of the species. *p-value < 0.05, **p-value < 0.01 and ***p < 0.001. PCoA, principal coordinate analysis; ANOSIM, analysis of similarities; HC, healthy controls; M, patients with metabolic dysfunction-associated fatty liver disease and RA, relative abundance.

To investigate the profile of the supragingival microbiota, we conducted metagenomic sequencing on the participants' supragingival plaque samples. The alpha diversity at the species level was determined with the Chao, Shannon and Simpson indices and no significant differences were observed between patients with MAFLD and healthy individuals (Figure 1A). Beta diversity was measured with the Bray‒Curtis distance, and the PCoA plot demonstrated the diverse microbial community structures between groups. A significantly lower Bray‒Curtis distance was detected in patients with MAFLD than in healthy individuals at the species level (Figure 1B and C).

Distinct differences in supragingival plaque microbiota composition were observed between patients with MAFLD and healthy individuals. The bacterial community in the supragingival plaque was composed predominantly of 20 bacterial genera. The top five most abundant genera were *Actinomyces*, *Corynebacterium*, *Neisseria*, *Capnocytophaga* and *Pseudopropionibacterium* (Figure 1D). Among the top 50 most abundant bacterial species, *A. naeslundii* was significantly enriched in the supragingival plaque from patients with MAFLD, whereas *Pseudopropionibacterium propionicum* (*P. propionicum*), *Cardiobacterium hominis* (*C. hominis*), *Capnocytophaga gingivalis* (*C. gingivalis*), *Capnocytophaga sputigena* (*C. sputigena*), *Capnocytophaga sp.* and *Porphyromonas catoniae* (*P. catoniae*) showed higher relative abundance in the healthy comparators (Figures 1E and S1A). Among the bacterial species with a relative abundance > 0.1%, eight species showed notably higher abundance in the disease group, whereas 18 species were significantly enriched in the healthy control group (Figure S1B). The co-occurrence network based on 26 taxa with differential abundance revealed that species enriched within the same group exhibited significant positive correlations with one another, and five *Firmicute* species formed a synergistic network in the supragingival plaque from patients with MAFLD (Figure 1F).

### Microbiota markers in supragingival plaque from patients with MAFLD

**Figure 2.**
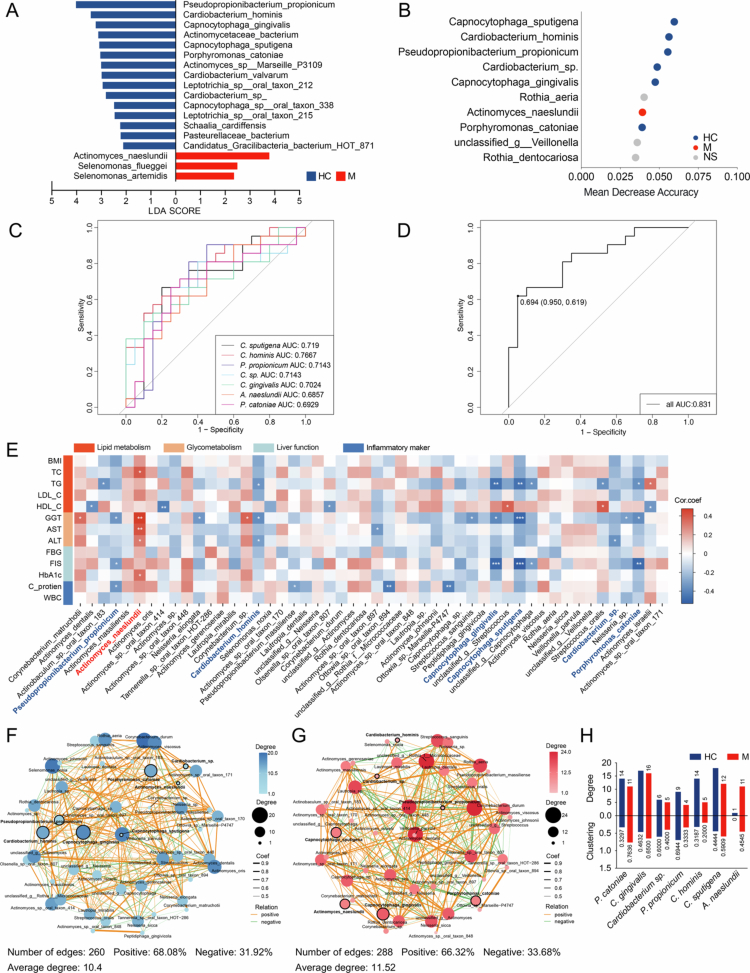
Microbial biomarkers in supragingival plaques. (A) Discriminative supragingival bacteria between the MAFLD and healthy groups identified by LEfSe analysis (LDA score > 2.0, *p*-value < 0.05, Wilcoxon rank-sum test). (B) The top 10 species that contributed most to the variations of supragingival microbiota between the healthy and MAFLD group as assessed by the mean decrease accuracy. The blue, red and grey colours indicate the significant enrichment of species in the healthy, MAFLD group and no significant different in abundance, respectively. (C) ROC analysis of 7 bacterial biomarkers. (D) Multivariate ROC analysis of seven bacterial biomarkers combination. (E) Heatmaps of Spearman's correlation coefficients between clinical parameters and the top 50 supragingival species. (F and G) Co-occurrence networks of the top 50 species in the healthy (F) and MAFLD (G) groups. The orange and green edges indicate positive and negative correlations, respectively (Spearman, *p*-value < 0.05, *R* > 0). The seven candidate biomarkers are labelled in bold, with nodes outlined in black. (H) The degree value and clustering value of seven bacterial biomarkers in the co-occurrence network of the healthy and MAFLD group. *p-value < 0.05, **p-value < 0.01 and ***p < 0.001. HC, healthy controls; M, patients with metabolic dysfunction-associated fatty liver disease; LDA, linear discriminant analysis; LEfSe, linear discriminant analysis effect size; ROC, receiver operating characteristic curve; AUC, areas under the curve; TC, total cholesterol; TG, total triglyceride; LDL-C, low-density lipoprotein cholesterol; HDL-C, high-density lipoprotein cholesterol; ALT, alanine aminotransferase; AST, aspartate aminotransferase; GGT, gamma glutamyl transpeptidase; FBG, fasting blood glucose; FSI, fasting serum insulin; HbA1c, glycated haemoglobin A1c; WBC, white blood cell; CRP, plasma high-sensitivity C-reactive protein and Coef, coefficient.

We further investigated potential bacterial biomarkers in supragingival plaque that distinguished patients with MAFLD from healthy comparators. LEfSe analysis was used to identify distinct microbial signatures with significant abundance differences across groups. The LDA scores indicated that three species, including *A. naeslundii*, predominated in patients with MAFLD, whereas 16 species were enriched in the healthy comparators (Figure 2A). A random forest model was used to identify the important features classifying MAFLD patients versus healthy individuals (Figures S2A and B). Among the top ten species with the highest mean decrease accuracy, seven species exhibited significant differences in abundance between groups (Figure 2B). Notably, *A. naeslundii* was the only species enriched in patients with MAFLD and thus may have potential implications in the condition. Meanwhile, six species, including *C. sputigena*, *C. hominis*, *P. propionicum*, *Cardiobacterium sp.*, *C. gingivalis* and *P. catoniae*, represented the core taxa in the supragingival plaque from healthy individuals. The efficacy of the seven candidates was further evaluated by ROC curve. The ROC-AUC for most candidates exceeded 0.700, and the integrative curve of the seven-species combination achieved an impressive ROC-AUC of 0.831, demonstrating the high differentiating performance of the oral microbiota in distinguishing patients with MAFLD from healthy comparators (Figure 2C and D).

Associations between the top 50 most abundant species and clinical parameters were assessed through Spearman's correlation analysis. As an oral biomarker for MAFLD, *A. naeslundii* demonstrated significant positive correlations with five disease indicators: TC, GGT, AST, ALT and HbA1c (Figure 2E). In contrast, six species characterising healthy comparators showed significant negative correlations with disease indicators (Figure 2E). Furthermore, to investigate the synergistic roles of the microbial consortia, we used a co-occurrence network combined with Spearman's correlation analysis, which indicated distinct microbiota interaction patterns between patients with MAFLD and healthy comparators. In healthy individuals, 260 significant pairwise interactions were observed among the top 50 species, with an average degree of 10.4 (Figure 2F). Only one significant correlation was observed between *A. naeslundii* and other species, whereas six health-associated species participated in 60 significant correlations with other community members (Figure 2H). In the MAFLD group, 288 significant pairwise interactions with an average degree of 11.52 were detected within the microbial community, thus indicating a more tightly interconnected network than that observed in healthy comparators (Figure 2G). Notably, the number of species exhibiting significant relationships with *A. naeslundii* increased to 11, and a pronounced negative correlation between *A. naeslundii* and *P. catoniae* was observed. Meanwhile, the total degree of the 6 health-associated species greatly reduced to 33, thus demonstrating the limited network of beneficial species (Figure 2H).

### Functional variations in the supragingival microbiota between MAFLD patients and healthy controls

**Figure 3.**
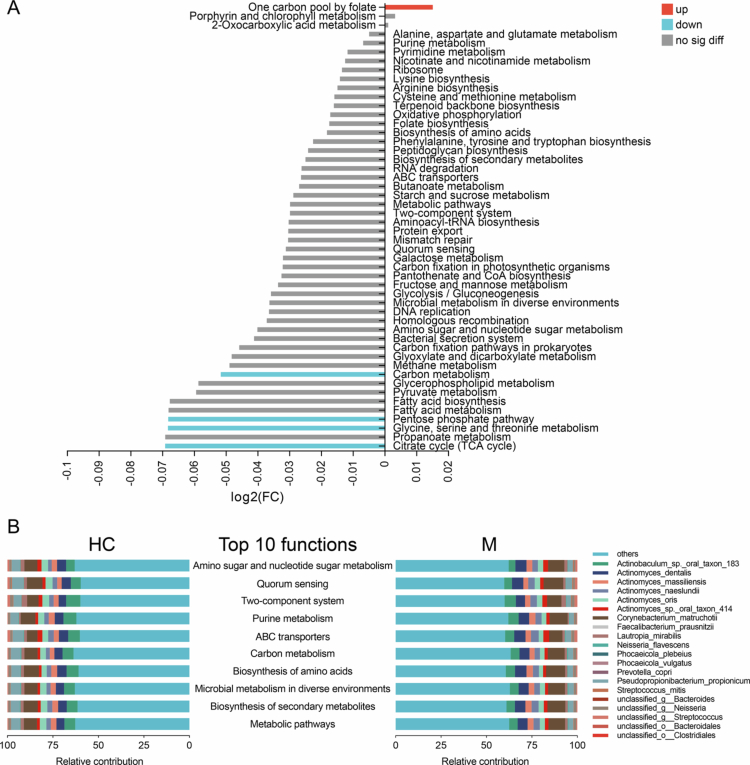
Functional variations in the supragingival microbiota between MAFLD patients and healthy controls. (A) Comparisons of the relative abundance of KEGG level 3 pathways between the healthy and MAFLD group, as shown by log2 fold change (Wilcoxon rank-sum test). The blue, red and grey colours indicate significant elevation of functional pathways in the healthy, MAFLD group and no significant different in abundance, respectively. (B) The top 20 species that contribute most to the top 10 most abundant functional pathways. HC, healthy controls; M, patients with metabolic dysfunction-associated fatty liver disease and FC, fold change.

The functional profiles of the supragingival microbiota were annotated with the KEGG database. Among the level 3 pathways with a relative abundance greater than 0.5%, the one carbon pool by folate pathway was significantly upregulated in the MAFLD group, whereas the carbon metabolism pathway; pentose phosphate pathway; and glycine, serine and threonine metabolism pathway were markedly downregulated (Figures 3A and S3A). The top ten most abundant functions were contributed primarily by *Corynebacterium matruchotii*, *Actinobaculum sp. oral taxon 183*, *Actinomyces dentalis*, *P. propionicum* and *Actinomyces oris* (Figure 3B). An examination of biomarker species indicated that the contribution of *A. naeslundii* to ten core functions was significantly higher in patients with MAFLD, whereas *P. propionicum* contributed more to the core functions in the healthy comparators (Figure S3B and C).

### Altered bacterial–fungal transkingdom interactions in supragingival plaques from patients with MAFLD

**Figure 4.**
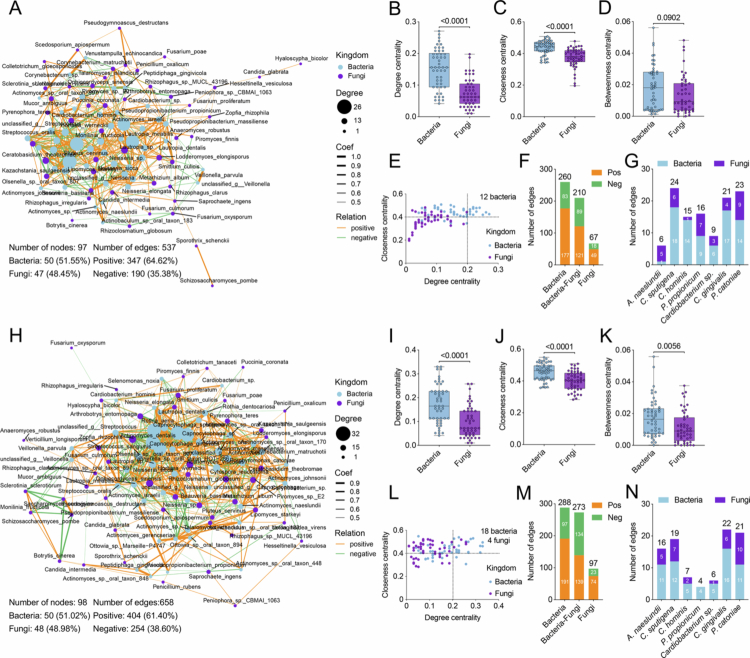
Bacterial‒fungal transkingdom interactions in supragingival plaques. (A and H) Transkingdom co-occurrence networks of the top 50 bacteria and the top 50 fungi in the healthy (A) and MAFLD (H) group. Orange and green edges indicate positive and negative correlations, respectively (Spearman, *p-*value < 0.05, *R* > 0). (B–D) Comparisons of node-level topological features (degree centrality, closeness centrality and betweenness centrality) of bacterial and fungal communities in the healthy group (Wilcoxon rank-sum test). (E) Degree centrality and closeness centrality values of each node in the healthy group. Nodes with a degree centrality value > 0.2 and a closeness centrality value > 0.4 are identified as core nodes. (F) Number of inter- and trans-kingdom edges of positive or negative correlations in the healthy group. (G) Number of inter- and trans-kingdom edges of seven bacterial biomarkers in the healthy group. (I–K) Comparisons of node-level topological features (degree centrality, closeness centrality betweenness centrality) of bacterial and fungal communities in the MAFLD group (Wilcoxon rank-sum test). (L) Degree centrality and closeness centrality values of each node in the MAFLD group. Nodes with a degree centrality value > 0.2 and a closeness centrality value > 0.4 are identified as core nodes. (M) Number of inter- and trans-kingdom edges of positive or negative correlations in the MAFLD group. (N) Number of inter- and trans-kingdom edges of seven bacterial biomarkers in the MAFLD group. Coef, coefficient; Pos, positive and Neg, negative.

In the past several years, the important roles of bacterial‒fungal transkingdom interaction in systemic disease have received increasing attention. Unique mycobiome patterns in supragingival plaque have been previously observed in patients with MAFLD by our research team [19]. We further investigated bacterial‒fungal interactions in the present study. The co-occurrence network with Spearman's correlation analysis revealed distinct transkingdom patterns in supragingival plaque between groups (Figure 4). Overall, the prevalent bacteria and fungi in supragingival plaques demonstrated a more frequent transkingdom communication in patients with MAFLD than in healthy comparators, as indicated by greater numbers of nodes and edges (Figure 4A and H). The bacterial community displayed a closer relationship than the fungal community in both patients with MAFLD and healthy comparators, as evidenced by a higher degree centrality, higher closeness centrality and lower betweenness centrality values (Figure 4B–D and I–K). In the network for the MAFLD group, 18 bacterial species and four fungal species were identified as core nodes (with a degree centrality value > 0.2 and closeness centrality value > 0.4) (Figure 4L), whereas only 12 bacterial species occupied core positions in the network for healthy comparators (Figure 4E). In addition, the network for the MAFLD group exhibited 273 significant bacterial‒fungal correlations (Figure 4M), whereas 210 transkingdom relationships were observed in the healthy group (Figure 4F). The interaction patterns of seven bacterial biomarkers were further investigated. The disease-associated *A. naeslundii* exhibited limited transkingdom interactions in healthy individuals, engaging with only six taxa (one bacterium and five fungi) (Figure 4G). In contrast, the network of *A. naeslundii* was substantially expanded in patients with MAFLD, involving 16 taxa (11 bacteria and five fungi) (Figure 4N). Regarding six health-associated species, a denser transkingdom network with a total of 108 significant relationships was observed in the healthy group, whereas the number of relationships was only 79 in patients with MAFLD (Figure 4G and N). These results elucidated the potential transkingdom oral dysbiosis in MAFLD.

### Oral administration of *A. naeslundii* aggravates MAFLD and disrupts intestinal microecological homoeostasis in mice

**Figure 5.**
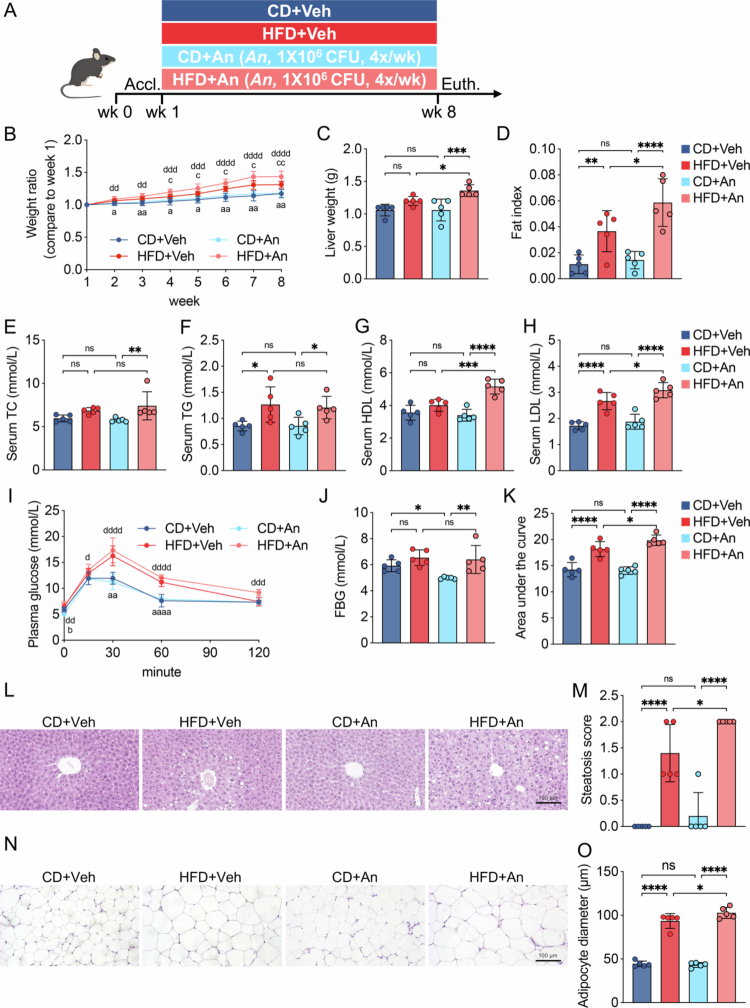
Oral administration of *A. naeslundii* aggravates MAFLD in mice. (A) Schematic illustration of the experimental design. (B) The weight ratio from week 1 to week 8. (C and D) Liver weights and fat indices at week 8. (E–H) Serum concentrations of TC, TG, HDL and LDL at week 8. (I) OGTT curve at week 7. (J) Fasting blood glucose values at week 7. (K) Areas under the OGTT curves at week 7. (L) HE staining of liver tissues. (M) Steatosis scores of liver tissues. (N) HE stainings of WAT tissues. (O) Adipose diameters of WAT tissues. Data in (C–H), (J and K), (M) and (O) are presented as mean ± SD. Comparisons were analysed using one-way ANOVA. The symbols a, b, c and d in (B) and (I) represent the significant differences between HFD + Veh vs CD + Veh, CD + An vs CD + Veh, HFD + An vs HFD + Veh and HFD + An vs CD + An, respectively. a, b, c, d and *p-value < 0.05; aa, cc, dd and **p-value < 0.01; ddd and ***p < 0.001; aaaa, dddd and ****p < 0.0001; ns, not significant. Accl, acclimation; Euth, euthanasia; wk, week; An, *A. naeslundii*; CD, chow diet; HFD, high-fat diet; Veh, vehicle; CFU, colony forming units; TC, total cholesterol; TG, total triglyceride; LDL, low-density lipoprotein; HDL, high-density lipoprotein; OGTT, oral glucose tolerance test; FBG, fasting blood glucose and WAT, white adipose tissue.

Since *A. naeslundii* was identified as a candidate oral biomarker for MAFLD, we further investigated its effects on the disease by establishing a MAFLD mouse model through giving a HFD accompanied by the oral administration of *A. naeslundii* (Figure 5A). The oral overabundance of *A. naeslundii* markedly promoted body weight gain in the mice fed a HFD. The weight ratio of the HFD + An group was significantly higher than that of the HFD + Veh group after the fourth week (Figure 5B). Furthermore, the significantly higher liver weight and fat index in the HFD + An group than in the HFD + Veh group indicated greater visceral lipid accumulation (Figure 5C and D). Accordingly, the oral administration of *A. naeslundii* aggravated lipid metabolism disorders triggered by HFD feeding, as evidenced by markedly elevated serum HDL and LDL in the HFD + An group (Figure 5E–H). Although no significant difference in fasting blood glucose was observed between the groups, the area under the OGTT curve was markedly higher in the HFD + An group than in the HFD + Veh group, thus demonstrating that *A. naeslundii* enhanced the severity of glucose tolerance impairment (Figure 5I–K). Furthermore, histological analysis revealed significant lipid accumulation, a higher hepatic steatosis score and a greater adipocyte diameter in the HFD + An group than in the HFD + Veh group (Figure 5L–O). These results indicated that the oral overabundance of *A. naeslundii* markedly aggravated MAFLD induced by HFD feeding in mice.

**Figure 6.**
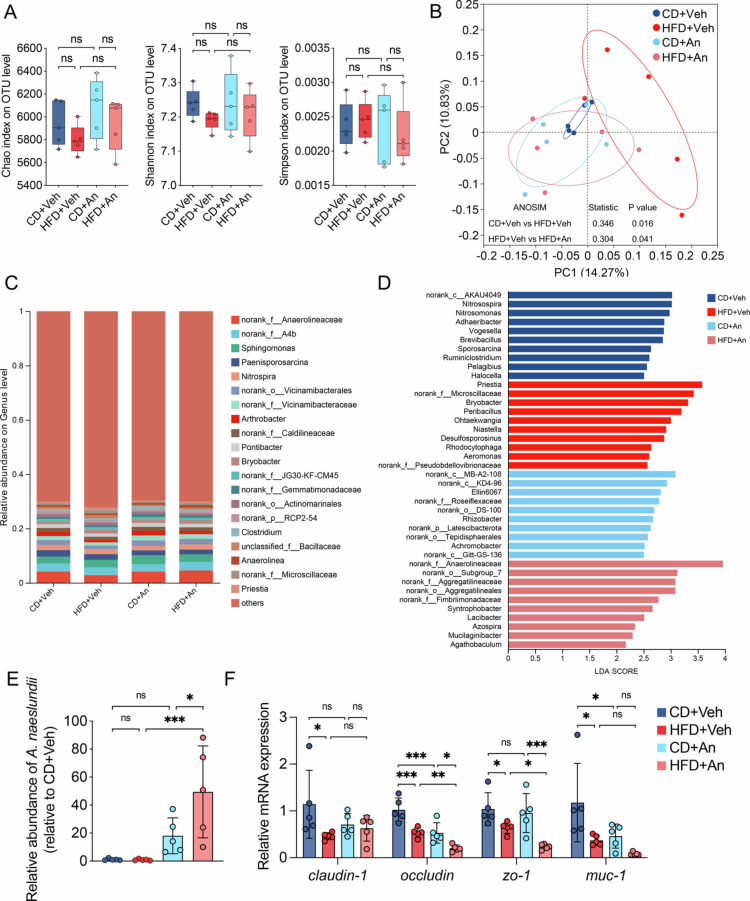
Oral administration of *A. naeslundii* affected the gut microbiota and intestinal tight junction protein in mice. (A) Alpha diversity assessed by the Chao, Shannon and Simpson indices at the OTU level (Kruskal‒Wallis H test). (B) PCoA with ANOSIM test based on Bray‒Curtis distance at the OTU level. (C) The composition of the supragingival microbiota at the genus level. The ‘other’ subcategory included rare species (relative abundance < 0.1%). (D) Discriminative gut microbiota of the four groups identified by LEfSe analysis (LDA score > 2.0, *p-*value < 0.05, Kruskal‒Wallis H test). (E) The abundance of *A. naeslundii* in faecal content quantified by qRT-PCR. Bacterial abundances are relative to CD + Veh group. (F) The relative gene expression levels of epithelial tight junction proteins in the ileum as quantified by qRT-PCR. Data in (E) and (F) are presented as mean ± SD. ns, not significant. PCoA, principal coordinate analysis; ANOSIM, analysis of similarities; An, *A. naeslundii*; CD, chow diet; HFD, high-fat diet; Veh, vehicle; LDA, linear discriminant analysis and LEfSe, linear discriminant analysis effect size.

Given the close connection between the oral cavity and the gut, disruption of the oral–gut axis might contribute to MAFLD development. Alterations of gut microbiota in mice were further investigated via 16S rRNA gene sequencing. Although no difference in alpha diversity was observed among the groups, ANOSIM indicated a statistically significant difference between the HFD + Veh group and the HFD + An group, thereby indicating notable alterations in the beta diversity of the gut microbiota caused by the oral overabundance of *A. naeslundii* (Figure 6A and B). Analysis of the gut microbiota composition indicated that the five most abundant genera were *norank_f_*_*Anaerolineaceae*, *norank_f_*_*A4b*, *Sphingomonas*, *Paenisporosarcina* and *Nitrospira* (Figure 6C). Distinct microbial signatures were revealed by LEfSe analysis and genera including *norank_f__Anaerolineaceae*, *norank_o__Subgroup_7*, *norank_f__Aggregatilineaceae*, *norank_o__Aggregatilineales*, *norank_f__Fimbriimonadaceae*, *Syntrophobacter*, *Lacibacter*, *Azospira*, *Mucilaginibacter* and *Agathobaculum* were significantly enriched in the HFD + An group (Figure 6D). These results indicated that the oral administration of *A. naeslundii* perturbed the gut microbiota balance. qRT-PCR revealed that a measurable amount of *A. naeslundii* in the faeces of mice in the CD + An and HFD + An groups, whereas the bacterium was nearly undetectable in the faeces of mice in the CD + Veh and HFD + Veh groups, suggesting the potential translocation of *A. naeslundii* through the oral–gut axis (Figure 6E). Furthermore, the gene expression levels of intestinal tight junction proteins, including Occludin, ZO-1 and MUC-1, were significantly downregulated in the HFD + An group compared to mice fed with HFD only group (Figure 6F), indicating increased gut permeability and disruption of the epithelial barrier induced by *A. naeslundii*.

## Discussion

MAFLD, fatty liver disease with the presence of metabolic dysfunction, affects approximately one-quarter of the population and poses a substantial global public health challenge, yet effective therapeutic targets remain lacking [21,22]. The roles of alcohol in the development of MAFLD has been well recognised, as it increases the NADH/NAD^+^ ratio, induces cytochrome P450 2E1 (CYP2E1) and generates toxic acetaldehyde [23]. Overnutrition is also a crucial factor contributing to hepatic steatosis, and dietary interventions, including calorie restriction, high-protein, low-carbohydrate diets and the intake of beneficial ingredients such as green tea, caffeine and coffee, are recommended to improve metabolic health [24,25]. In recent years, extensive evidence has elucidated the crucial roles of the gastrointestinal microbiota in MAFLD onset and progression [25,26]. Given that the oral microbiota constitutes the second largest microecosystem in the human body, the association between oral microbiota dysbiosis and MAFLD has received increasing attention in recent years. Our previous study documented alterations in the oral mycobiome in patients with MAFLD and suggested potential links of oral microorganisms to MAFLD [19]. The present study established a clinical cohort and collected supragingival plaque samples to investigate oral microbiota profiles in patients with MAFLD. This study specifically targeted the supragingival microbiota, as supragingival plaque serves as a crucial ecological niche for oral bacteria. Metagenomic sequencing revealed altered diversity, composition and correlation network in the oral bacterial community of patients with MAFLD. Further animal studies indicated that the oral overabundance of *A. naeslundii*, which was identified as a candidate biomarker for the disease, was associated with disrupted gut homoeostasis and worsened metabolic dysfunction in mice.

Community diversity and composition analysis revealed apparent oral dysbiosis in patients with MAFLD. In line with findings from previous studies [27], we detected differences in the beta diversity of the oral microbiota between patients with MAFLD and healthy individuals. The diminished oral microbial beta diversity in the MAFLD group suggested the homogenisation of species, poorer ecological restoration capacity and a narrower functional spectrum than those observed in the healthy comparators. Among the 26 discriminative species with a relative abundance >0.1%, all five *Firmicutes* species were enriched and constituted a synergetic network in the MAFLD group. The accumulation of gut *Firmicutes* and elevated *Firmicutes*/*Bacteroidetes* ratios are well-recognised characteristic microbial alterations in individuals with metabolic dysfunction, including obesity, fatty liver disease and type 2 diabetes mellitus (T2DM) [28–30]. Associations between oral *Firmicutes* enrichment and T2DM have also been documented [31,32], and the current study extend these observations by revealing enrichment and co-occurrence of *Firmicutes* species in the supragingival plaques of MAFLD patients. This parallel variation between oral and gut ecosystems points to potential interactions along the oral–gut axis.

Co-occurrence network analysis can unravel interaction patterns and provide comprehensive insights into microorganism assembly and community structures [33]. While altered gut microbial networks in individuals with metabolic disorders have been reported, findings remain inconsistent. Cheng et al. observed enhanced gut network robustness following lifestyle interventions, coinciding with the alleviation of fatty liver disease [33], whereas Wu et al. reported homogeneous interaction patterns and increased connections in the gut microbial community in nonalcoholic fatty liver disease [34]. In the current study, the MAFLD group exhibited intensified interactions among the top 50 most abundant oral species, characterised by increased correlation numbers and a higher network degree compared to healthy controls. Additionally, individuals with MAFLD also exhibited an expanded oral bacterial‒fungal transkingdom network, as evidenced by more core members and correlation edges. The transkingdom dysbiosis in the gut microecosystem have been linked to systemic diseases such as T2DM and obesity [35,36], whereas altered transkingdom interactions in the oral microecosystem have been reported primarily in patients with localised oral diseases, such as early childhood caries and periodontitis [37,38]. Thus, the disrupted oral network pattern observed in the current study may implicate the role of the oral microbiota in metabolic disease. Furthermore, our findings revealed interspecific connection patterns involving the feature species. *A. naeslundii* was strongly associated with MAFLD severity and exhibited enhanced connections with both bacterial and fungal species in the MAFLD group. The expansion of opportunistic pathogens might be attributable to the expansion of *A. naeslundii*. In summary, alterations in community interactions reflected the oral ecological dysregulation in MAFLD.

The oral microbiota has been investigated for its potential to provide non-invasive diagnostics in oral diseases, including periodontitis, caries, early childhood caries and oral squamous cell carcinoma [39–41]. Studies have also detected the high predictive performance of oral microorganisms in systemic diseases, such as colorectal cancer, autism spectrum disorder and metabolic dysfunction [19,42,43]. The findings in current study support the discriminatory capacity of seven resident bacteria as candidate biomarkers for MAFLD. Among these biomarkers, *A. naeslundii* was uniquely enriched in the disease group. *Actinomyces* are facultatively pathogenic commensal organisms found in the human alimentary tract [44,45]. Previous studies have also observed the enrichment of *Actinomyces* spp. in the oral cavity and gut of patients with chronic liver disease, as well as the predictive capability of *A. naeslundii* for MAFLD [46–48]. Although the specific pathogenic mechanisms of *Actinomyces* spp. remain unclear, their links to inflammatory processes have been proposed [44,49]. By establishing a MAFLD mouse model through orally administering *A. naeslundii*, we found that the oral overabundance of *A. naeslundii* significantly aggravated MAFLD under the HFD condition.

Given the close connection between the oral cavity and the gut, the impact of oral dysbiosis on the gut microecosystem was further investigated. Previous studies on the oral–gut–liver axis have focused mostly on periodontal pathogens, such as *P. gingivalis*, which aggravated MAFLD by destroying gut barrier functions, elevating serum inflammation markers and altering gut metabolic pathways [5,15,18]. The current study provided additional evidence that the oral–gut axis in exacerbating MAFLD, as the oral administration of *A. naeslundii* resulted in its faecal detection in mice. This potential translocation of oral bacteria may contribute to dysbiosis of the gut microecosystem and disruption of the gut epithelia barrier, which promoted the MAFLD progression [5,15,25,50]. Notably, our findings suggest that, beyond pathogenic oral species, the overgrowth of oral commensal bacteria may also contribute to gut dysbiosis, highlighting the importance of maintaining oral homoeostasis.

Several limitations should be considered when interpreting the findings of this study. First, the sample size of the clinical cohort was not sufficiently large, which may limit the generalisability of the results. More participants should be enroled in future study to mitigate potential confounding factors. Second, detailed records of participants' nutritional status and oral health were not available, restricting the ability to adjust for relevant confounders. However, all participants were recruited from the same regional population with a shared dietary pattern, and none reported extreme dietary habits, which reduced concerns about substantial dietary variation influencing the observed microbial differences. Although the clinical metadata were inexhaustive regarding oral health indicators such as the plaque index or gingival index, all the participants met the screening criteria and exhibited no significant oral health abnormalities. Future studies should incorporate comprehensive and standardised assessments of diet and oral hygiene to definitively disentangle the effects of these factors from disease-specific oral microbiome signatures. Moreover, future research should explore the potential correlation between oral and gut microbial communities. Finally, while the current study observationally demonstrated potential exacerbating roles of *A. naeslundii* in MAFLD, the underlying mechanisms remain to be elucidated and require further in-depth exploration.

## Conclusions

In conclusion, the current research revealed oral dysbiosis in patients with MAFLD. Compared with healthy individuals, patients with MAFLD exhibited altered diversity, composition and functions in the supragingival microbiota, along with more extensive interbacterial and bacterial‒fungal transkingdom interactions. Furthermore, seven resident bacteria were identified as candidate biomarkers with high predictive performance in discriminating patients with MAFLD from healthy individuals. *A. naeslundii*, the sole candidate species enriched in the MAFLD group, was linked to gut dysbiosis and more severe MAFLD in mice when orally administered under HFD feeding. The current research established significant associations between oral microbiota and metabolic dysfunction, providing novel insights into oral prevention and treatment of MAFLD.

## Supplementary Material

Supplementary material Supplemental_figures_and_figure_legends_.docx

Supplementary material Supplemental table and legend.docx

## Data Availability

All data generated or analysed during this study are included in this article.
