# Supplementary material for: Supragingival Actinomyces naeslundii aggravates metabolic dysfunction-associated fatty liver disease via the oral–gut axis
Source: J Oral Microbiol. 2026 Mar 7;18(1):2639208. doi: 10.1080/20002297.2026.2639208 (PMC12973853; doi:10.1080/20002297.2026.2639208)
Supplement: Supplementary material — Supplemental_figures_and_figure_legends_.docx [file ZJOM_A_2639208_SM4771.docx]

**Supplemental Figures and Figure legends**


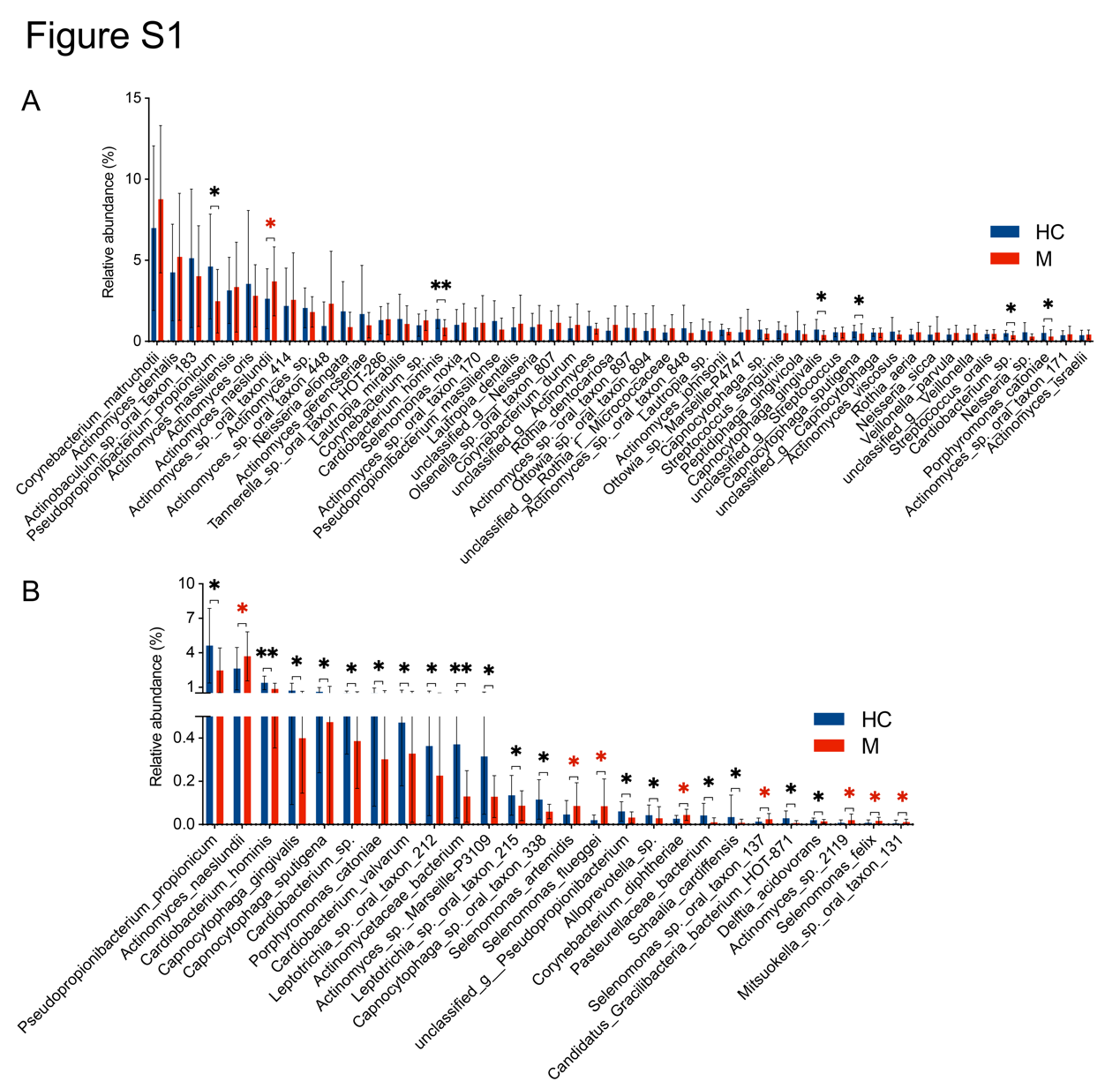


**Figure S1.** Statistical differences of supragingival microbiota between the healthy and the MAFLD group. **(A)** Comparisons of relative abundance of top 50 species (Wilcoxon rank-sum test). **(B)** Comparisons of relative abundance of species with relative abundance > 0.1% (Wilcoxon rank-sum test). *P value < 0.05, **P value < 0.01. HC, healthy controls; M, patients with metabolic dysfunction-associated fatty liver disease.


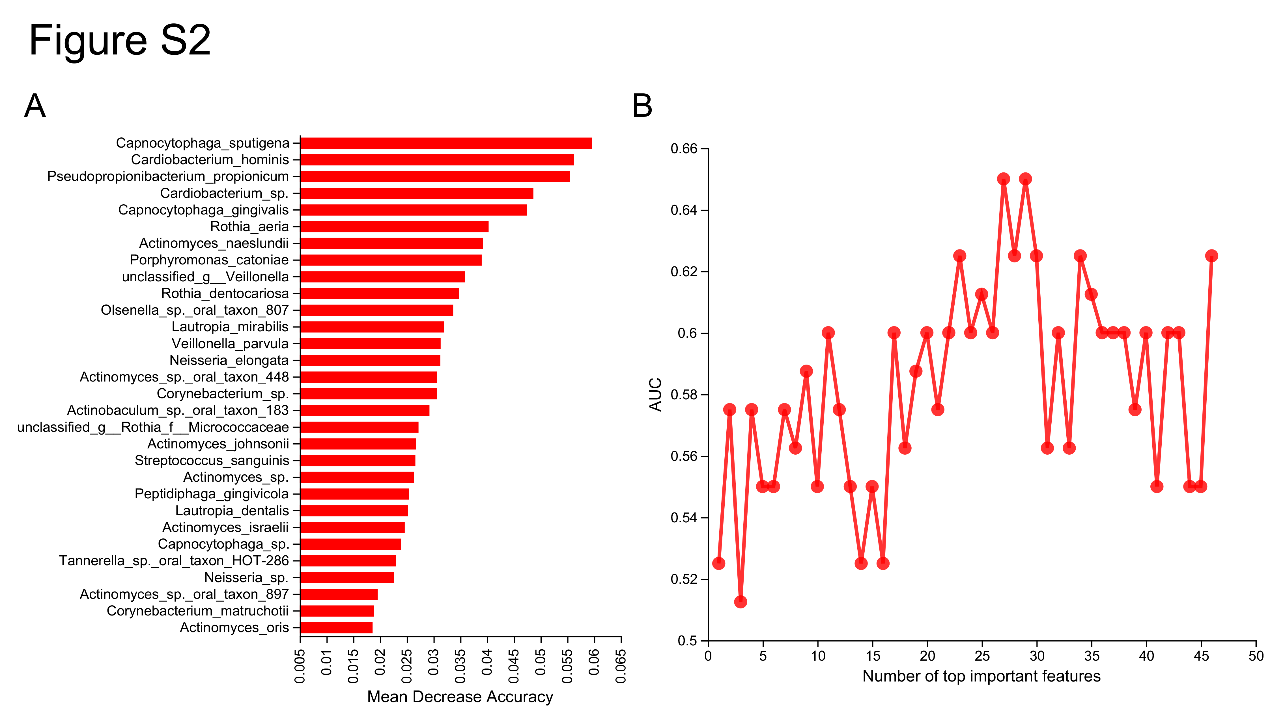


**Figure S2.** The construction of the random forest model for identifying candidate oral biomarkers. **(A)** Bar plot of the variable importance of species with high mean decrease accuracy. **(B)** AUC evaluation by using the top important feature. AUC, areas under the curve.


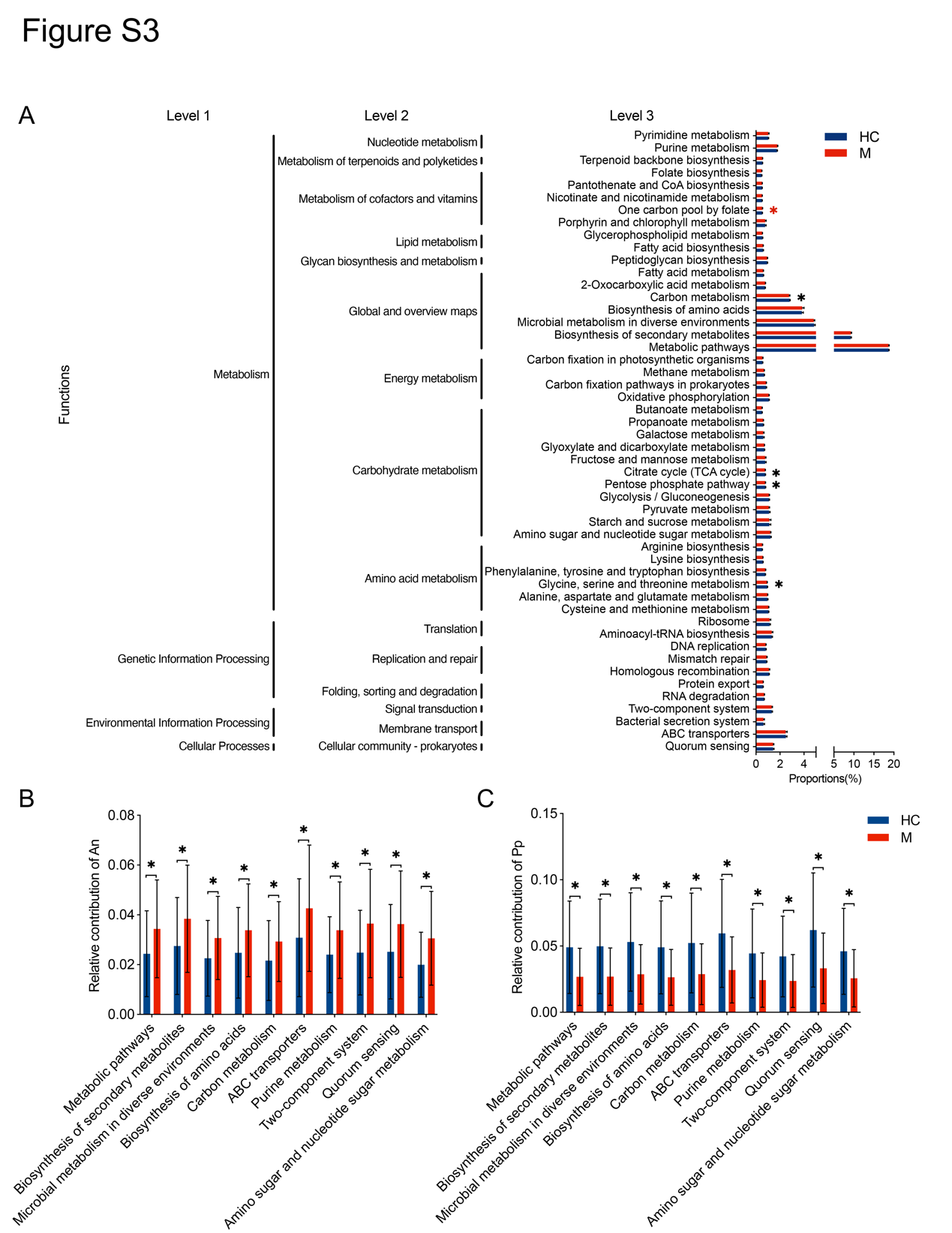


**Figure S3.** Functional variations in the supragingival microbiota between MAFLD patients and healthy controls. **(A)** Comparisons of the relative abundance of KEGG level 3 pathways between the healthy and the MAFLD group. Significant differential abundance between groups is indicated by black asterisks (healthy group) and red asterisks (MAFLD group) (Wilcoxon rank-sum test). **(B)** Comparisons of contributions of *A. naeslundii* to ten core functions (Wilcoxon rank-sum test). **(C)** Comparisons of contributions of *P. propionicum* to ten core functions (Wilcoxon rank-sum test). *P value < 0.05. HC, healthy controls; M, patients with metabolic dysfunction-associated fatty liver disease, An, *A. naeslundii*; Pp, *P. propionicum*.
