# Supplementary material for: Supragingival Actinomyces naeslundii aggravates metabolic dysfunction-associated fatty liver disease via the oral–gut axis
Source: J Oral Microbiol. 2026 Mar 7;18(1):2639208. doi: 10.1080/20002297.2026.2639208 (PMC12973853; doi:10.1080/20002297.2026.2639208)
Supplement: Supplementary material — Supplemental table and legend.docx [file ZJOM_A_2639208_SM5420.docx]

**Supplemental Table 1.** Primers for quantitative real-time PCR analysis

| **Primers** | **FP (5’ to 3’)** | | **RP (5’ to 3’)** | |  |
| --- | --- | --- | --- | --- | --- |
| *gapdh* | GGGTGTGAACCACGAGAAAT | | CCTTCCACAATGCCAAAGTT | |  |
| *occludin* | GGCAAGCGATCATACCCAGAG | | AGGCTGCCTGAAGTCATCCAC | |  |
| *claudin-1* | AGCTGCCTGTTCCATGTACT | | CTCCCTTTGTCTGCTGCTC | |  |
| *zo-1* | CCACCTCTGTCCAGCTCTTC | | CACCGGAGTGATGGTTTTCT | |  |
| *muc-1* | AGTTACGGTCAGGCTGCTCCGTGGT | | ACCCTCCCGGAAAACCACAGTC | |  |
| *16s-univ-1* | GGTGAATACGTTCCCGG | TACGGCTACCTTGTTACGACTT | |  |  |
| *A. naeslundii* | ATCGTGGTGCTCATGTTCGT | TGAGCGAGTATGGAATCGGC | |  |  |
